# Cell Cycle Deficits in Neurodegenerative Disorders: Uncovering Molecular Mechanisms to Drive Innovative Therapeutic Development

**DOI:** 10.14336/AD.2019.0923

**Published:** 2020-07-23

**Authors:** Chitra Joseph, Abubakar Siddiq Mangani, Veer Gupta, Nitin Chitranshi, Ting Shen, Yogita Dheer, Devaraj KB, Mehdi Mirzaei, Yuyi You, Stuart L Graham, Vivek Gupta

**Affiliations:** ^1^Faculty of Medicine and Health Sciences, Macquarie University, Sydney, NSW 2109, Australia; ^2^School of Medicine, Deakin University, Melbourne, VIC, Australia.; ^3^Department of Molecular Sciences, Macquarie University, North Ryde, NSW 2109, Australia; ^4^Save Sight Institute, Sydney University, Sydney, NSW 2109, Australia

**Keywords:** Cell cycle, Apoptosis, Neuron, Neurodegeneration, CDK, Cyclin

## Abstract

Cell cycle dysregulation has been implicated in the pathogenesis of neurodegenerative disorders. Specialised function obligates neuronal cells to subsist in a quiescent state of cell cycle once differentiated and therefore the circumstances and mechanisms underlying aberrant cell cycle activation in post-mitotic neurons in physiological and disease conditions remains an intriguing area of research. There is a strict requirement of concurrence to cell cycle regulation for neurons to ensure intracellular biochemical conformity as well as interrelationship with other cells within neural tissues. This review deliberates on various mechanisms underlying cell cycle regulation in neuronal cells and underscores potential implications of their non-compliance in neural pathology. Recent research suggests that successful duplication of genetic material without subsequent induction of mitosis induces inherent molecular flaws that eventually assert as apoptotic changes. The consequences of anomalous cell cycle activation and subsequent apoptosis are demonstrated by the increased presence of molecular stress response and apoptotic markers. This review delineates cell cycle events under normal physiological conditions and deficits amalgamated by alterations in protein levels and signalling pathways associated with cell-division are analysed. Cell cycle regulators essentially, cyclins, CDKs, cip/kip family of inhibitors, caspases, bax and p53 have been identified to be involved in impaired cell cycle regulation and associated with neural pathology. The pharmacological modulators of cell cycle that are shown to impart protection in various animal models of neurological deficits are summarised. Greater understanding of the molecular mechanisms that are indispensable to cell cycle regulation in neurons in health and disease conditions will facilitate targeted drug development for neuroprotection.

Cell cycle is an organized and meticulously timed series of cellular events that leads to the duplication of genetic material. Cell cycle comprises multiple processes that are broadly classified into interphase, mitosis or M phase and cytokinesis. Specific cell cycle checkpoints tightly regulate the progression of cell through these various stages. The cumulative process of cell cycle is made possible by a group of regulatory proteins, cyclins and cyclin-dependent kinases (CDKs) [1]. A specific cyclin/cdk interaction results in the activation or disengagement of downstream molecules that determines the next stage of cell cycle progression. Cyclin D and CDK4/6 interaction during G1/S phase transition for instance, phosphorylates retinoblastoma protein (pRb) thereby inducing reversal of the transcriptional repression on E2 Transcription Factor (E2F) and directing cell division process. Cyclin E/CDK2 complex appears in the later stages of G1/S phase and adjudicates the propriety of cell progression to further division or differentiation pathways [2, 3]. It is at this stage that checkpoint kinases from either cip/kip (CDK interacting protein/Kinase inhibitory protein) or CDK4 inhibitory modules of INK4a/ARF contribute to the regulatory events of cell cycle affirming cell-fate decisions. p21, p27, and p57 belong to the cip/kip family of inhibitors that are activated by p53 and transforming growth factor beta (TGF-β) in response to DNA damage [2, 4]. The INK4a/ARF inhibitors p16 and p14 further regulate G1/S transition. p16^INK4A^ inhibits CDK4 driven pRb phosphorylation whereas p14^ARF^ impairs cell cycle by promoting stable binding with murine double minute 2 (MDM2), which is a p53 inhibitor whose binding promotes p53 mediated apoptotic pathways [5]. This eventually leads to cyclin A replacing cyclin E association with CDK2 and directs the cells to S phase. S phase involves duplication of genetic material during which cyclin A associates with CDK1 and as it progresses to G2 phase with cyclin B gradually replacing cyclin A. Cyclin B/CDK1 complex formation helps cellular entry into M phase, with subsequent degradation of cyclin B which is essential for cells to exit mitosis. Other major classes of regulatory proteins involved in the cell cycle are the cell division cycle (cdc) family of proteins. These proteins participate in multiple stages of cell cycle regulation by assisting CDK activation *via* modulation of inhibitory partner phosphatases [6].

Neurons constitute the basic structure of nervous system and chronic neuronal impairment induced by cell cycle dysregulation might impede various brain cognitive, behavioural, motor and regulatory functions. Incongruous cell cycle re-entry, eventually leading to apoptotic activation has been suggested to play a pathological role in various neurodegenerative conditions. Pathological accumulation of neurotoxic assemblies of β-amyloid, p-tau, parkin, α-synuclein have been implicated in abnormal cell cycle activation in differentiated neurons [7] This review provides comprehensive insights into the biochemical processes associated with cell cycle regulation in neuronal cells and discusses the potential implications of their dysregulation in the onset and progression of neuropathological events.

## Cell cycle dysregulation and disease involvement

A balance in cellular proliferation and cell death mechanisms ensures cell and tissue homeostasis is maintained. Dysregulation of this intricate network may result in defective cell cycle causing disease. Aberrant cell cycle may either cause cells to attain unlimited proliferative potential as likely observed in neoplastic, pro-inflammatory and auto-immune disorders or may trigger persistent cell loss as recorded in a host of neurodegenerative, cardiovascular and auto-immune pathologies. A deleterious mutation in the Fas death receptor leading to defective T lymphocyte apoptosis was showed to be involved in altered cell cycle regulation in autoimmune diseases [8]. AIDS, another autoimmune disease has also been associated with faulty cell cycle regulation. Contagious apoptosis phenomenon was evident in HIV-1 Env expressing cells under stress conditions that might be involved in transmitting apoptotic signals to healthy CD4^+^ bystander cells [9]. In the case of Myocardial infarction, cardiomyocyte loss was partially restored upon overexpressing cyclins and CDKs [10]. Comparably, distinct types of cancer cells have been shown to harbour mutations affecting almost all aspects of the cell cycle regulation [11-13]. Accordingly, sustained efforts have been made to establish various CDKs and CDKIs as diagnostic and prognostic markers as well as drug targets in management of various kinds of cancers [13]. More recently, defective cell cycle regulation has emerged as an apparent feature of several neurodegenerative disorders, manifested by chronic neuronal cell loss.

## Cell cycle control in neurons

Neuronal cells along with other cell types such as muscle cells are unique in that these remain quiescent once they exit the cell cycle due to their terminally differentiated nature. Cell cycle regulatory proteins in neural cells continue to be required for axonal migration, maturation and regulating synaptic plasticity [14]. One or more of these cell cycle proteins and pathways might get activated in response to various epigenetic or pathological stimuli. For example, cyclin-c mediated retinoblastoma protein phosphorylation and G0 exit activates non-homologous end joining (NHEJ) repair mechanism [15]. Park et al (1998) demonstrated the role of cyclin-dependent kinases (CDK) and cyclin-dependent kinase inhibitors (CKIs) in DNA damage evoked neuronal death. DNA damaging agents like UV irradiation, Ara-C and camptothecin driven apoptosis in primary rat sympathetic and cortical neurons was rescued upon overexpression of p27, p16 and CDK4/6 using Sindbis virus. Elevated levels of cyclin D1 in cortical neurons treated with camptothecin further substantiated the protective role of CDK4/6 [16]. Similar protective functions of CKIs and CDK4/6 on postmitotic neurons deprived of nerve growth factor (NGF) have been reported previously [17].

Camptothecin treatment of cortical neurons was effective in elevating the phospho pRb levels - a key feature of DNA damage-induced cell death [18]. Ajioka et al. (2007), highlighted the ability of differentiated neurons to evade death and actively replicate *via* p107, a member of Rb family of proteins. In the mouse retina, inner nuclear layer (INL) cells were able to proliferate and clonally expand with a single copy of p107 [19]. p107 phosphorylation is a regulated cell cycle event mediated by D-type cyclins which is found to induce differentiation in nerve growth factor (NGF) treated PC12 cells [20, 21]. Nerve growth factor stimulated Rb negative retinal ganglion cells (RGCs), were shown to exist in a tetraploid state as a consequence of aberrant mitosis [22].

An interesting study in the developing chick embryo showed a cell type-specific pattern of mitotic cell fate in two different cell populations. Undifferentiated cells from two different regions of developing chick embryo follow discreet cell fates post proliferation. Cells from ciliary ganglia acquire the potential to terminally differentiate after completing mitosis whereas sympathetic ganglion cells retain their ability to divide post-differentiation [23]. This shows that neuronal sub type plays a major role in defining cell cycle fate.

## Aberrant cell cycle activation and apoptosis in neuronal cells

Cell cycle regulatory genes play a prominent role in programmed cell death (PCD) and there is evidence suggesting their role as contributing factors to neuronal apoptosis. Cell cycle re-entry by neurons is a prerequisite to apoptotic pathway activation that constitutes a key underlying molecular feature of most neurodegenerative disorders. Cell cycle reactivation is a protective mechanism adapted by the cells in response to chronic injury, but the exact mechanistic basis of apoptosis onset remains ill-defined. Neurotrophic factor deprivation could be one of the contributory factors and postmitotic neurons deprived of NGF indeed were shown to exhibit selective induction of cyclin D1. Upon NGF withdrawal, neuronal cells exhibit programmed cell death features associated with global decline in RNA and protein synthesis with concomitant decrease in cell cycle-related gene expression suggesting that onset of neurodegenerative processes might be a result of the abortive cell cycle [24]. DNA damage-inducing agents are similarly known to trigger anamolous cell cycle activation. Differentiated PC12 neuronal cells, sympathetic neurons, and cerebral cortical neurons were rescued from a DNA damaging agent camptothecin induced death by G1/S inhibitors and CDK inhibitor treatments [25].

Further studies suggest that toxic β-amyloid oligomers might play a role in inducing atypical cell cycle stimulation. In this regard, CDK4/6 was shown to be involved in β-amyloid induced cell death in differentiated PC12 cells and rat cortical neurons. CDK4/6 driven apoptosis was linked with increased phosphorylation of pRb/p107. CDK inhibitor flavopiridol treatment, as well as E2F/DP inhibition, was able to impart protection to neuronal cells against β-amyloid toxicity suggesting the involvement of CDK4/6 linked pathways in β-amyloid induced neuronal apoptosis [26]. Interestingly, a link between cell cycle regulators and apoptotic pathway activation was shown in animal model harbouring G-protein-regulated inward-rectifier potassium channel 2 (GIRK2) missense mutations. Cerebellum staining in these mice was positive for cell cycle proteins such as proliferating cell nuclear antigen (PCNA), CDK4, cyclin D and cyclin A, although no evident proliferative events could be observed. Upregulation of these markers was consistent with the decreased levels of CDK inhibitor p27 in cells of the external germinal layer [27]. Apoptosis in post mitotic neurons in the developing rat brain was also observed to be triggered by BAD phosphorylation mediated by cell cycle protein cdc2 [28].

The role of another major cell cycle regulator protein and apoptosis marker p53 in the neuronal apoptosis has been of great interest in the field. Rats treated with excitotoxic glutamate analogue, kainic acid induced p53 expression in damaged cells and was characterized by condensed nuclei and eosinophilic cytoplasm. Interestingly, treatment with protein synthesis inhibitor cycloheximide could decrease the p53 induction in these apoptotic neurons [29]. Aberrant cell cycle induction precedes cell death events as observed in postmitotic neurons of both central (CNS) and peripheral nervous system (PNS) of mice embryos expressing mutant Rb protein. Increased p53, p21, and cyclin E levels were evident in dying cells of CNS under these conditions; however, PNS exhibited p21 induction independent of p53 leading to cell death [30]. Other studies suggest that cell death activation in mature neurons might be linked to defective DNA damage response. Increased levels of Cdc25A, a G1 to S phase cell cycle regulator and indicator of cell cycle activation were observed in rat cortical neurons exposed to DNA damaging agents. Furthermore, a critical cell cycle checkpoint kinase ataxia-telangiectasia mutated protein (ATM) dysregulation was found to be responsible for genotoxic agent-induced cell cycle activation [31] suggesting that apoptosis could potentially trigger atypical cell cycle activation in these non-dividing neuronal cells.

## Factors triggering cell cycle activation in neurons

Cell cycle regulatory proteins are well expressed in neurons and play roles independent of proliferation related functions such as synapse formation and dendritic development [14]. Pathological and epigenetic events can induce proliferation coupled functions of cell cycle proteins and precipitate the cellular crisis by activating cell-division associated biochemical pathways in cells that are otherwise quiescent. This aberrant signal to divide may arise from one or more of the altered biochemical processes in cells. Proteolytic imbalance of cell cycle regulators can for instance hamper signalling pathways essential for axonal guidance and neural wiring [32]. Altered proteolytic cleavage and post translational events may also cause toxic accumulation of protein aggregates in cells [33, 34]. Protein aggregates such as TDP-43 and C9ORF72 repeats in amyotrophic lateral sclerosis (ALS) [35], polyQ-expanded huntingtin in HD [36], hypo-phosphorylated tau and amyloid β in AD [37], α-synuclein in parkinson's disease (PD) [38] are generally removed via proteasomal machinery but excessive toxic overload of aggregates may expose cells to persistent oxidative stress. Among the multiple inducers of DNA damage in neurons, reactive oxygen species (ROS) play a major role as brain is the largest consumer of oxygen. ROS are capable of generating modified bases and single strand breaks (SSBs) [39]. Accumulation of unrepaired damaged DNA is widely observed in aged cells due to deficiencies in cellular repair machinery. Defective DNA damage repair mechanisms and oxidative stress might collectively contribute to revival and reactivation of cell cycle proteins leading to *faux pas* of cell-awakening event. Neurotrophin signalling similarly can trigger abnormal mitogenic signalling that ends up in stalemate with imminent nucleic acid duplication but cells being unable to process cytokinesis. This eventually triggers activation of apoptotic pathways via p53, pRb and E2F1 cell-cycle regulatory proteins [40].

### Proteolytic control of cell cycle

Synchronization between specific proteases and cell cycle inhibitory factors regulates smooth and irreversible progression of cell division. A sustained induction of autophagy and endocytosis paralleled by a progressive decline in proteolytic function of lysosomes adversely affects the normal cell-cycle regulation in neurons [6]. Defective lysosomal degradation promotes cell death processes as neurons are unable to get rid of misfolded proteins. Protein aggregates associated with neurodegenerative disorders may trigger alterations in cell cycle and induce apoptotic changes [41]. Interestingly, reversible protein aggregation was shown to be a protective mechanism in the restart of cell cycle after exposure to stress [42] and regulate cyclin transcription and localization [43]. Ectopic cell cycle re-entry mediated through oligomeric amyloid beta (Aβ) and tau-phosphorylation has been observed [41] in a significant fraction of the neurons affected in Alzheimer’s disease (AD) [44]. Amyloid β is a normal by-product of amyloid precursor protein (APP) metabolism and requires Aβ-degrading proteases for effective clearance of the peptide from cells [41, 45]. Large bulk of such accumulated proteotoxins are removed by autophagy; a natural degradation pathway essential to remove damaged proteins and organelles. An intact TORC1-*SCH9* pathway signal from a mature vacuole has been identified for cell-cycle progression and the loss of a functional vacuole may trigger specific arrest of cells in early stages of G1 phase [46]. Autophagic vacuoles have been detected in dystrophic neurites in human and mouse AD brains. Not surprisingly, restoration of normal lysosomal function in mice models of AD depicted promising therapeutic effects on neuronal function [16]. A direct link between dysregulated Cdk5 mediated autophagy disruption was evident in the death of dopaminergic neurons as an age dependent mechanism [47]. Vacuolar protein sorting 34 (vps34), a protein that interacts with Beclin 1 and initiates autophagy is inhibited by Cdk1 and Cdk5 mediated phosphorylation at Thr159. This inhibitory phos-phorylation plays a major role in membrane trafficking and toxic degradation that in turn could promote neurodegenerative processes [48]. A major control of cell cycle is also mediated through interactions of cell cycle proteins with caspases, many of which have been implicated in regulating neuronal apoptosis. Activated caspases induce proteolytic processing of p27, a key nuclear inhibitor of cell cycle progression, leading to its stabilisation and subsequent cell cycle exit [49]. Animals subjected to selective ablation of various caspases demonstrate noticeable neuronal phenotypes, with caspase 3 and 9 impaired animals exhibiting prominent deficits in neuronal development [50]. Consequently, caspase dysregulation has been implicated in Alzheimer’s, ALS and Huntington’s disease pathologies [51]. Involvement of caspase-7 and/or caspase-3 in regulating the mitotic cell cycle progression was recently demonstrated by its inhibition using siRNAs [52]. Cyclin A1 levels were shown to be suppressed by DNA damage induced caspase-1 expression [53]. Cell cycle proteins cdk1/cyclin B1 in turn regulate caspases by promoting Thr125 phosphorylation that inhibits apoptotic initiation and regulates mitosis [54]. A prominent switch of cell cycle control is also regulated through the ubiquitin proteasome system [55]. Ubiquitin-like-specific protease 1 (Ulp1) is indispensable for cell-cycle and mutations have been associated with compromised cell cycle progression. Sumoylation adds small ubiquitin-like modifiers (SUMOs) to proteins and sentrin-specific protease 5 (SENP5) was shown to be involved in removing SUMOs from sumoylated proteins and alter the protein function [56]. Inhibition of SENP5 led to an increase in cells with more than one nucleus thus demonstrating the involvement of this protease in cell division [57]. Two major classes of ubiquitin ligases, the Skp, Cullin, F-box containing complex (SCF complex) and the APC/C-Cdh1, play pivotal roles in the cell cycle regulation through the ubiquitination of various cell cycle proteins. Dysfunction of the SCF box protein complex for instance, is implicated in Huntington’s [58] while APC/C-Cdh1 downregulation is involved in erroneous cell cycle re-entry in AD [59]. Accumulating evidence indicate that dysfunction of the ubiquitin mediated proteolysis may be central to some of the pathological features observed in neurodegenerative diseases. Overall, dysregulated proteostatic homeostasis may breach cellular compliance maintained through caspases, ubiquitin-proteasome system and lysosomal function and encourage re-entry of the neurons into an ineffective cell cycle, leading to neuronal loss in disease conditions [60, 61].

### DNA damage response

There are specific checkpoints at G1/S, G2/M and anaphase that act as quality control and initiate cell-cycle arrest upon identification of damage. Ataxia telangiectasia and Rad3-related protein (ATR) and ataxia telangiectasia-mutated (ATM) along with two downstream kinases, checkpoint kinase 1 and 2 (Chk1 and Chk2) mediate DNA damage response (DDR) that culminates in either cell cycle arrest or DNA repair. However, DNA damage repair machinery itself is susceptible to defects that can promote accumulation of DNA lesions. Mutations in DNA polymerase the enzyme involved in DNA synthesis, for instance, hamper its proofreading capacity affecting sequence fidelity. Oxidative damage and subsequent by-products like lipid peroxides, aldehydes etc further induce DNA damage [62]. Involvement of G1 phase cell cycle components in oxidative stress induced DNA double strand breaks show cell cycle activation as an essential event in DNA damage repair in neurons [63]. Each cell has specific repair machinery to deal with different types of lesions and prevent or minimise the damage from being reproduced. Robust DNA damage response (DDR) is crucial for the genome integrity and non-replicating cells require a constitutively active DDR machinery for sustenance [64]. Nucleotide excision repair (NER), base excision repair (BER), mismatch repair (MMR), homologous recombination (HR) and non-homologous end joining (NHEJ) are some of the major biochemical processes that play a protective role in neuronal cells [65]. NER and BER are the major repair pathways in neurons activated in response to oxidative DNA damage. With ageing, there is increasing accumulation of unrepaired DNA lesions due to defective repair mechanisms. The neurons harbouring DNA lesions may transcribe faulty proteins that can result in perpetuating and amplifying the genetic error or damage.

Simpson et al (2015) evidenced accumulation of DNA lesions and defective DDR in aging brains. DNA damage related molecules γH2AX and DNA-dependent protein kinase- catalytic subunit (DNA-PKcs) positive neurons have been demonstrated in the brains of the dementia patients [66]. NHEJ and HR pathway markers were also observed in mouse model of AD with impaired repair mechanisms associated with increased DNA damage. Further, imbalance in DDR was observed when the mice were fed on a high-fat diet indicating the possible link between obesity and faulty DDR in Alzheimer’s [67]. NHEJ and HR pathways are mainly associated with repair of DNA double strand breaks. As homologous DNA sequence is a pre-requisite for HR mediated actions it is primarily activated in the G2/M phase of cell cycle where as NHEJ repair is effective throughout all the stages of cell cycle and mediates re-ligation of broken DNA strands.[68] Suberbielle et al. (2013) showed transient increase in DNA double-strand breaks (DSBs) with increased brain activity that was exacerabted with β-amyloid accumulation [69]. Interestingly, breast cancer associated gene 1 (BRCA1) depletion was also implicated in failed DNA repair machinery in human AD brains as well as hAPP transgenic mice [70]. BRCA1 is a DNA damage repair factor that helps maintain genome stability *via* mainly NHEJ and NER repair pathways. BRCA1 deficiency like NHEJ affects almost all checkpoints involved in cell cycle and induce apoptosis in response to DNA damage. BRCA1 interacts with multiple cell cycle and check point kinases like cyclins, CDKs, p53 and ATM that makes it a key molecule in cell cycle regulation during DNA damage [71]. Mounting evidence suggests that mitochondrial DNA damage in the substantia nigra of Parkinson’s brain appear much before clinical signs of neuronal degeneration are evident [72, 73]. Sepe et al. (2016) further confirmed the role of impaired NER in dopaminergic neurons *in* an Ercc1 ablated mouse model [74]. All these repair mechanisms in post-mitotic neurons suggest cell cycle activation as a crucial event in neuronal DDR [75]. Oxidative stress which is one of the major factors implicated in DNA damage also plays a role in spinal cord pathology associated with sporadic and familial forms of ALS. These ALS spinal cord neurons were positive for OH8dG, an oxidative DNA damage marker [76]. FUS mutated mouse model of ALS exhibited DNA damage characterised by increased γH2AX staining [77]. Farg et al. (2017) recently reported DDR as an inducible factor in C9orf72 repeat formation, a widely reported genetic aberration in ALS patients [78]. All these scenarios involving DNA damage promote re-entry of neurons to cell-cycle and subsequent neuronal death at G1/S phase [75].

### Oxidative stress and Cell cycle

Unregulated reactive oxygen species (ROS) generation, reduced ROS scavenging, impaired mitochondrial function and antioxidant defence mechanisms induce oxidative stress that might lead to altered processing of cellular signals. ROS has been implicated in regulating cell cycle progression through its effects on the molecular processes that regulate cyclin/CDK complexes [79] and mitochondrial stability. ROS is implicated in promoting mitochondrial DNA mutations and inducing mitochondrial dysfunction through its detrimental effects on mitochondrial respiratory chain proteins [80]. Mitochondrial dysfunction in turn, negatively affects ATP production, impairs Ca^2+^ homeostasis and induces apoptotic pathway activation [81]. AMP/ATP ratio is increased as a consequence of reduced ATP levels, that activates AMP-activated protein kinase (AMPK). AMPK stimulation elicits cell cycle defects at G1/S cell cycle checkpoint preventing the transition of cells from G1 to S phase [82]. Human brain glioma cells U251 were indeed shown to undergo G1 arrest as a mitochondria-mediated apoptosis mechanism transactivating caspase-3 and caspase-9 proteins [83]. Lee et al. (2014) identified a link between morphodynamic mitochondrial modifications and subsequent cell cycle arrest. Accordingly, human mitochondrial fission 1 protein (hFis1) depleted cells displayed remarkably elongated mitochondria and augmented expression of G2/M phase regulatory proteins cyclin A, cyclin B1, CDK1, polo-like kinase1 (Plk1), aurora kinase A and Mad2 that are involved in G2/M arrest [84].

ROS generation during normal cellular physiology can be toxic, once the cellular threshold of antioxidant mechanisms is breached. Recent research has revealed increased vulnerability of dopaminergic neurons to oxidative stress. Klein et al. (2002) described correlation between oxidative stress and cell cycle re-entry causing neurodegeneration in a Harlequin (hq) mutant mouse model. Hq mutation disrupts apoptosis inducing factor expression (Aif), a mitochondrial oxidoreductase enzyme that serves as a free radical scavenger. Cell cycle dysregulation was further substantiated with positive PCNA and cdc47 staining in Hq mutant cerebellar and retinal neurons [85]. Lee et al. (2008) established mitogenic pathway activation in response to ROS generated by neurotoxin bisphenol A (BPA; 2,2-bis-(4 hydroxyphenyl) propane). BPA exposure induced dysregulation of intracellular Ca^2+^ levels and stimulated downstream transcription factors like MAPK, NF-κB and growth factor receptors thus initiating cell cycle re-entry in post-mitotic neurons [86]. Rat cortical neurons exposed to H_2_O_2_ mediated oxidative stress exhibited DNA double strand breaks and subsequent G1 stage activation as demonstrated by increased pRb and cyclin D1 levels. Incubation with H_2_O_2_ redirected the cells to apoptosis via S phase re-entry which could be attenuated with CDK4/6 and CDK2 inhibition using siRNAs. These reports suggest cell cycle re-entry as an apoptotic triggering event aggravated by oxidative stress induced double strand breaks (DSBs) [63]. Similarly, H_2_O_2_ induced oxidative stress in PC12 cells that were differentiated with NGF, showed mitochondrial dysfunction and elevated cyclin D1 and pRb levels indicating molecular activation of cell cycle machinery [87].

Furthermore, auto-oxidation of dopamine generates ROS and other free radicals that may play a pathological role in PD. Studies have shown oxidative DNA damage in leucocytes in PD patients [88]. In retina, oxidative damage has been shown to be a major component of degenerative disorders such as glaucoma [89], retinopathies [90], and AMD [91] pathologies. Retina is rich in polyunsaturated fatty acids (PUFAs) including docosahexaenoic acid (DHA) and their oxidation leads to increased ROS generation. In AD, amyloid and tau aggregation constitute a hallmark pathological feature that disturbs ROS equilibrium and contributes to elevated ROS levels. Increased ROS in turn is disruptive for mitochondrial function and has been widely implicated in the neurodegenerative condition [92].

### Neurotrophins and cell cycle

**Figure 1.**
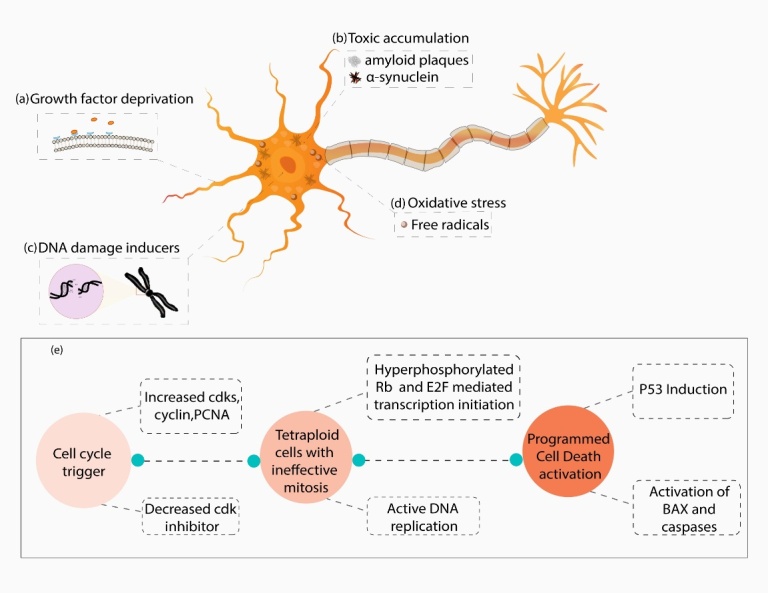
**Schematic representation elucidating various factors involved in cell cycle activation and apoptosis induction in neurons**. Growth factor deprivation (a), neurotoxic accumulation of protein polymers (b), DNA damage (c) and oxidative stress (d) may lead to aberrant cell cycle activation characterised by an increase in cyclin-dependent kinases (CDKs), cyclin, proliferating cell nuclear antigen (PCNA) and a decrease in CDK inhibitors. The synchronised effects of increased cdk4/6, cdk2 and decreased CDK inhibitors p21 and p16 (e) result in retinoblastoma protein (Rb) hyperphosphorylation and release of E2 Transcription Factor (E2F) that initiate transcription of target genes responsible for DNA replication. However, due to lack of mitotic signals (activation of cyclin A and cell division cycle 2 (cdc2)), these actively replicating cells are unable to exit the cell cycle and end up with double the amount of DNA. This leads to p53 induced apoptosis activation, and bax, caspase upregulation that drives the neurons to programmed cell death (PCD).

Nerve growth factor (NGF), brain-derived neurotrophic factor (BDNF), neurotrophin 3 (NT-3), and NT-4/5 are major constituents of neurotrophin family and regulate survival, growth and differentiation of neurons by binding to two types of receptors, the Trk tyrosine kinases receptors (TrkA, TrkB and TrkC) and the non-tyrosine kinase p75 neurotrophin receptor (p75^NTR^). Neurotrophins affect a spectrum of key signalling networks that are intricately linked with cell cycle regulation which establishes these as important modulators of cell fate in neural pathophysiology [93-95]. Mitogenic effects of neurotrophins are evident with the induced proliferation potential of cells affected upon binding to respective Trk receptor. P75^NTR^ is widely restricted to antimitogenic responses based on its relative ratio to Trk receptors. p75^NTR^ activates the small GTPase, RhoA which is involved in cytokinesis through its association with Rho kinase (ROCK) and citron kinase [96]. Rho is also found to be the governing factor restricting cyclin D1 expression to mid-G1 phase of cell cycle via sustained ERK activity [97]. p75^NTR^ on interaction with NGF is reported to activate proto-oncogene Rac that further induces cyclin D1 expression triggering mitogenic effects [98, 99]. Remarkably, cell cycle proteins p53, pRb, and E2F1 showed altered subcellular localisation and phos-phorylation profile in human neuroglial cultures that were treated with BDNF and NGF. These altered cell cycle events act as the driving factor towards neurodegenerative changes observed in association with increased neurotrophic factors [40]. In contrast, animal gene knockout studies have shown that the absence of BDNF, NT-3 and their receptors TrkB and TrkC result in cerebellar dysfunction, increased apoptosis and altered neuronal connections [100, 101]. BDNF plays an important role in cell-cycle regulation as its treatment inhibited cell cycle re-entry in primary cortical neuronal cultures stressed with excitotoxic and oxidative stress and induced neuroprotection. Pharmacological inhibition experiments revealed that these effects of BDNF were mediated through both PI3K-Akt and Ras-MAPK pathways. BDNF treatment did not reduce the ER stress marker CHOP levels, suggesting that BDNF protective effects were independent of its modulatory effects on ER stress pathways [102]. BDNF silencing similarly resulted in cell cycle arrest at G0/G1 phase in B-type lymphoma cells and promoted activation of apoptotic proteins like Bax, activated caspase-3 and caspase-9 and cleaved poly (ADP-ribose) polymerase (PARP) under in vitro conditions [103]. In the retina, low affinity receptor p75^NTR^ binding to endogenous NGF leads to loss of retinal neurons [104]. An intracellular interactor of the p75^NTR^ called brain-expressed X-linked 1 (Bex 1) was identified as a link between the neurotrophin signalling and cell cycle [105]. Different neurotrophins are shown to differentially affect specific pathways in various neuronal populations. For instance, all neurotrophins except NGF were found to promote the survival and/or differentiation of calbindin-immunopositive and GABAergic striatal neurons [106]. Another study reported the promotion of central cholinergic and dopaminergic neuron differentiation by BDNF but not NT-3 [107]. Summarising, different factors discussed here, have a regulatory effect on neuronal re-entry to cell cycle and subsequent apoptosis induction as an important converging feature of various neurodegenerative processes (Fig. 1)

**Table 1:** Cell cycle modulatory proteins/genes identified in Alzheimer’s disease (AD), Parkinson’s disease (PD), amyotrophic lateral sclerosis (ALS) and ophthalmic neurodegenerative disorders such as glaucoma, age related macular degeneration (AMD) and diabetic retinopathies are listed along with the tissues examined.

Diseases	Gene	Region	Cell cycle phase	Refs
Alzheimer's disease	CDK1/Cdc2, Cyclin B1	Neurons with neurofibrillary tangle s (IHC)	G0-G1, G2/M	[108]
	Cdk5	Whole brain lysate (WB)	S	[109]
	p25/CDK5	Neuro fibrillary tangle bearing neurons (WB, IHC)	G1/S	[110]
	cyclin B1, cyclin D, CDK4, PCNA	Hippocampus, subiculum, locus coeruleus, dorsal raphe, inferotemporal cortex, cerebellum	G1/S, M	[111]
	Cyclin D1, cyclin E, Cyclin A pRb	Cortical neurons exposed to β amyloid	G1/S	[112]
	CDK2, CDK4, CDK6, cyclin B, and cyclin D	Peripheral lymphocytes (WB)	G1/S, M	[113]
	CDK4, p16	Hippocampus (IHC)	G1	[114]
	P25, p35	Frontal cortex, inferior parietal cortex and hippocampus (WB)	G1/S	[115]
	CDC25A	neurofibrillary tangles and senile plaque bearing neurons Hippocampus (WB, IHC)	G1/S	[116]
	Cyclin D, PCNA, Cyclin B1	Hippocampus (IHC)	G1/S, M	[108]
	Cdc2/Cyclin B1, CyclinD1, CDK4, pRb PCNA	Hemibrain homogenates	G1/S, M	[117]
		Hippocampus (WB, IHC)	G1/S	
	BAX	Senile plaques Hippocampus (IHC)		[118]
	P53	superior temporal gyrus (WB)	G1	[119]
	Rb	neurofibrillary tangles and senile plaque bearing neurons (IHC) Hippocampus	G1/S	[120]
Glaucoma & other retinal disorders	Gadd45a and Ei24(p53 family)	Retina (qPCR and WB)		[121]
	Cyclin D1	Optic nerve head (ONH) (Microarray)	G1	[122]
	CDKN2A & CDKN2B	Retina (RT-PCR)	G1	[123]
	Cyclin B1	RPE cells (WB)	G2/M	[124]
	p53, p21	RPE cells (WB)	G1	[125]
	p16	Primary RPE cells (WB)	G1	[126]
	CDKN1	RPE cells (WB)	G1	[127]
	NUCKS1	peripheral human retina (PR) and peripheral RPE-Choroid-Scleral (PRCS) tissues (RNA-Seq)	M	[128]
	Cyclin D	Macular cells (qRT PCR)	G1	[129]
	Bax	Retina (WB & IF)	G1	[130]
	p27Kip1	Retina (IHC)	G1	[131]
	p53	Retinal pericytes (WB)	G1	[132]
Amyotrophic lateral sclerosis	E2f, cyclin D1, CDK4, pRb	lumbar spinal cord and pre-/postcentral gyrus (IHC, WB)	G1	[133]
	Bax, caspase 3, caspase 8, p53, ppRb	lumbar spinal cord (IHC, IF, WB)	G1	[134]
	P53	Spinal cord, motor cortex (IHC, WB)	G1	[135]
	CDK5	Spinal cord (IHC, WB)	G1/S	[136]
	CDK4, CDK5, CDK6, cyclin D1	Spinal cord (IHC)	G1	[137]
Parkinson's disease	Rb	SN nuclei, Frontal cortex and hippocampus (IHC)	G1/S	[138]
	PCNA, E2F,	Dopaminergic neurons in SN (IHC, IF)	G1/S	[139]
	PCNA,	Dopaminergic neurons in SN(IF)	G1/S	[140]
	cyclin D, cyclin A, cyclin E and cyclin B	IF of embryonic rat mid brain neurons	G1/S, G2/M	
	CDK5, CDK2	Dopaminergic neurons in SN (IHC, WB, kinase assay) in MTP treated mice	G1/S	
	p53	caudate nucleus (WB) in human PD brain	G1	[141]
	p53	Dopaminergic cells (IF, WB) in vitro (6-hydroxydopamine) and mouse model (PQ/MB) Whole brain lysates (WB) in human PD brains	G1	[142, 143]

## Dysregulated cell cycle in various neurodegenerative disorders

Neurodegenerative disorders are invariably associated with increased apoptosis with growing evidence of altered biochemical processes linked with cell cycle regulation. Here we discuss the involvement of aberrant cell cycle activation in representative neurodegenerative disorders including AD, ALS, PD and retinal disorders like glaucoma, age-related macular degeneration and diabetic retinopathy. A list of proteins and genes that are associated with cell cycle dysregulation in those neurodegenerative disorders and tissue examined are tabulated below (Table 1).

### Alzheimer's disease

AD is a condition of progressive cognitive impairment leading to dementia with an increased susceptibility in advanced age. Individuals present with differing patterns of disability and have different progression rates with fast to slow deterioration of neuronal cells. A diverse range of environmental, genetic and life style associated factors contribute to disease onset. Although the exact mechanisms underlying AD development remain unclear, accruing evidence has suggested protein malfunction in the brain leading to series of toxic molecular events as a key culprit. AD pathology is characterised by cognitive decline and other neurodegenerative changes such as senile plaque and neurofibrillary tangle (NFT) formation in the brain [144].

Neurons are terminally differentiated in adult CNS and remain in a quiescent G0 state of cell cycle under normal conditions. However, in AD, evidence suggests that there is dysregulation of biochemical pathways in neurons that might affect cell cycle regulation [145]. Increased levels of cyclins, CDKs, altered mitochondrial activity and hyperphosphorylated tau have been extensively observed in the neurons in AD [146]. Positive staining was observed for cyclin B1, cyclin D, CDK4 and PCNA in degenerating neurons from post-mortem AD subjects, inferring region specific changes in cell cycle regulatory components in the disease [108]. Amyloid β peptide treatment of the cortical neurons similarly showed substantially increased markers of the S-phase in the neuronal population along with increased cyclin D1, cyclin E, cyclin A and phospho Rb levels. These were subsequently identified to undergo apoptosis upon prolonged Aβ treatment and interestingly some of the apoptotic effects of Aβ were recoverable upon treatment with pharmacological inhibitors of cell cycle [147]. Studies suggest the involvement of CDKs in causing dysregulated cell cycle events in AD pathology. CDK1 activation leading to dysregulated neuronal functions revealed co-localisation of CDK1/cyclin B1 complex in the neurofibrillary tangles [148]. Amyloid β was shown to promote mTORC1 signalling and CDK2 mediated tau phosphorylation leading to increased neuronal degeneration [149]. Further, in a study conducted on lymphocytes from AD patients Kim et al. (2016) reported significant to moderate upregulation of CDK2, CDK4, CDK6, cyclin B, and cyclin D cell cycle proteins compared to the samples from normal subjects [113]. An aberrant colocalization of CDK4 and p16 proteins in AD brains compared to age matched controls has been observed [150]. Other studies show CDK5 upregulation and p25 accumulation in AD [115]. p25 is a truncated form of p35, a regulatory subunit essential for CDK5 activation in the neuronal cells under normal conditions. The p25/CDK5 complex was found to augment tau hyperphosphorylation and p25/CDK5 expressing neurons exhibited increased cytoskeletal disruption and formation of apoptotic cell bodies [151]. Thus, an imbalance in cell cycle proteins could potentially be an indication of early stages of AD pathology and contribute to disease progression. Increased Cdc25A immunoreactivity consistent with an increase in Cdc2/cyclin B kinase activation has been observed in neurofibrillary tangles and senile plaques in AD tissues [116]. Hippocampal pyramidal neurons in AD were observed to be positive for cyclin D and mitotic marker PCNA and in contrast, cells in nucleus basalis were observed to be positive for PCNA [108].

Consistent with the data from human samples, animal models of AD also exhibit dysregulated cell cycle proteins in the CNS neurons. Park et al. (2007) developed a simian virus 40 large T antigen (TAg) expressing mouse model that exhibited augmented cyclinD1, CDK4, pRb and PCNA levels compared to the wild type counterparts, suggesting biochemical activation of the cell cycle modulatory pathways. Progressive neurodegenerative changes following abrupt cell cycle activation were evidenced by condensed chromatin, cleaved caspase 3 and formation of vacuolar structures in cortex and hippocampus regions. Animal models of AD demonstrate tau hyperphosphorylation and amyloid plaque formation, the two hallmark histopathological features of disease [117]. Upregulation of microtubule binding protein tau has also been shown to contribute to abnormal cell cycle events. Nuclear localisation of cyclin E is crucial to its role in regulating DNA replication ensuring S-phase progression and 1N3R isoform of tau promoted cyclin E translocation from the nuclei to cytoplasm. Expression of 1N3R isoform in HEK293 cells accordingly induced S phase arrest with a reduced cell number in G1 and G2/M phases [152]. The exact mechanisms underlying tau pathology and cell cycle events in AD are not very clear. Hyperphosphorylated retinoblastoma protein (ppRb), a key regulator for G1/S transition, was however, shown to correlate with tau hyperphosphorylation in the 3×Tg mouse model of AD. Both early and late markers of tau protein alterations were observed to colocalize with ppRb in the brain samples from human AD subjects, suggesting mechanistic links between aberrant cell cycle re-entry and tau pathology [153]. Overexpression of human tau in drosophila similarly impaired mitosis through its microtubule binding domain with spindle malformations, leading to aneuploidy and apoptotic cell death. These effects were primarily mediated through its inhibitory effects on the kinesin Klp61F function, drosophila homologue of kinesin-5 protein [154].

Additionally, ploidy studies on AD patient samples revealed evidence of abnormal chromosomal content indicative of an erroneous cell cycle machinery. DNA content analysis in the hippocampus and basal nucleus of human AD post-mortem brain samples showed increased number of polyploid cells [44, 155, 156] reflecting active DNA replication but an inability to proceed to M phase [157]. Increased pro-apoptotic proteins have consistently been reported in AD pathology. For example, hippocampal AD neurons and AD patient brain tissues show upregulated expression of bax [158] and p53 proteins that have been implicated in apoptotic pathway activation and tau phosphorylation respectively [119]. Rb, a key cell cycle regulator was also identified to undergo extensive phosphorylation in tissues from AD patients. Hyperphosphorylated Rb interestingly, was primarily localized with neurofibrillary tangles and neuritic plaques suggesting its potential crosstalk with tau and Aβ in determining aberrant cell cycle awakening events in disease conditions [120].

### Glaucoma and age-related retinal disorders

Glaucoma is a neurodegenerative disorder affecting the visual pathway, primarily the retinal ganglion cells (RGC), that shares overlap of molecular features with AD such as chronic and progressive neuronal loss along with Aβ and tau protein deposition [159, 160]. AD patients exhibit some retinal nerve fibre layer thinning and reduced RGC density which are also characteristic features of glaucoma [161, 162]. Experimental models of optic nerve injury have revealed apoptotic bodies and condensed nuclei in RGCs. DNA fragmentation in RGCs, one of the early events associated with apoptosis depicts positive correlation with intraocular pressure (IOP) increase [163]. TUNEL positive cells are also present in human eye tissues from open-angle glaucoma subjects suggesting RGC death by apoptosis [164]. Gene array analysis from high IOP and optic nerve transection rats indicated upregulation of apoptotic genes. qPCR and western blotting further revealed p53 pathway represented by Gadd45a and Ei24 to be enriched. Significantly upregulated levels of CDK2 were also observed in both optic nerve transection and experimental glaucoma retinas suggesting involvement of cell cycle regulatory mechanisms [121]. Transcriptomics studies on ocular hypertensive rat model displayed up-regulated cyclin-D1 levels and other cell cycle genes [122, 165]. CDKN2B-AS1 is a long non-coding RNA (lncRNA) that has been identified in several genome wide association studies carried out on various cohorts of glaucoma patients [123, 166, 167]. Cyclin-dependent kinase inhibitor 2B (CDKN2B) and cyclin-dependent kinase inhibitor 2A (CDKN2A) encode cell cycle proteins p16INK4A, p14^ARF^and p15INK4B respectively. Upregulated CDKN2A and CDKN2B levels in the retina of ocular hypertensive rat model demarcate the association of cell cycle protein changes with RGC and axonal loss observed in experimental glaucoma [123].

Age-related macular degeneration (AMD) is another neurodegenerative disorder that mainly affects retinal pigment epithelium (RPE) leading to neovascularisation and loss of photoreceptors at the late stage of the disease [168]. RPE is a specialised monolayer of pigmented cells situated between retina and choroid and is involved in several functions essential for retinal physiology and photoreceptor excitability [169, 170].

Cell cycle dysregulation was evident in H_2_O_2_ induced oxidative stress in RPE cells with increased cyclin-B1 levels driving early events of mitosis [124]. Oxidative stress induced bone morphogenetic protein-4 expression in RPE was shown to mediate senescence *via* activation of cell cycle molecules p53 and p21 [125]. Mammalian target of rapamycin (mTOR); a master regulator of proliferation and cell growth mediated processes, drives cultured human derived primary RPE cells to replicative senescence and induce upregulation of CDK inhibitor p16^Ink4a^ [126]. A study in ARPE-19 cell culture showed that death-associated protein like-1 (DAPL1) maintained the quiescent state of RPE cells and restored cell proliferative events through CDK inhibitor 1 (CDKN1) [171]. Transcriptomic profiling of AMD retinas further revealed upregulation of nuclear ubiquitous casein and cyclin-dependent kinase substrate 1(NUCKS1), which acts as a substrate for CDK1 during mitosis [128]. In addition, elevated mRNA expression levels of cyclin-D as a proliferation associated altered Wnt signalling pathway regulation was reported in both animal models and macular tissues from AMD patients [129].

Diabetic retinopathy (DR) is a complication of systemic diabetes and affects the retinal vasculature with evidence of apoptosis of retinal neurons and widespread vascular complications [172]. Apoptotic marker, Bax upregulation was observed in post-mortem diabetic retinas, and similar findings were found in bovine retinal pericytes exposed to high glucose [130]. Retinal pericytes are at an increased risk of loss under hyperglycemic conditions. This early loss of retinal pericytes was linked to post-translational O-linked β-N-acetylglucosamine (O-GlcNAc) modification of cell cycle regulator p53 [132]. Pleiotrophin, a neurotrophic factor [173] is shown to be involved in cell proliferation associated with diabetic retinopathy. Increased pleiotrophin levels were observed in vitreous and epiretinal membrane tissues from proliferative diabetic retinopathy patients. Interestingly, cell cycle arrest was also rendered in ARPE19 cells through pleiotrophin silencing, that mainly affected the cells in G0/G1 and S phases of cell division [174].

### Amyotrophic lateral sclerosis

ALS is characterized by progressive degeneration of nerve cells involved in voluntary muscle movements. There is sequential loss in patient ability to move with initial difficulties observed in movement to progressive impairment in speech and respiration. Although majority of the ALS cases are reported to be sporadic there is increasing evidence supporting the familial onset and the association of genes like TDP43, SOD1, C9orf72 in contributing towards increased susceptibility [175].

Disease progression involves multiple levels of disrupted molecular network with cell cycle dysregulation suggested to be one amongst them [133]. Several cell cycle regulatory molecules with altered expression profile have been observed in cell culture and ALS conditions *in vivo*. Rb is a negative regulator of cell cycle events and activation of Rb blocks the G1 to S phase transition by binding to the E2F promoter. Hyperphosphorylated Rb promotes release of E2F that assists in transcriptional activation and this pathway has been shown to be negatively affected in ALS [133]. Post-mortem analysis of spinal cord samples revealed that although the DNA binding ability of E2F remained unaltered there was a significant difference in the subcellular localization of E2F that might be indirectly involved in the apoptotic pathway activation in ALS. Atypical E2F accumulation across various regions was attributed to the increased levels of cyclinD1, CDK4 and hyperphosphorylated Rb [133]. Further the differential roles of p53 in ALS spinal cord motor neurons but not in the motor cortex region were also observed. Co-localisation studies revealed positive staining of p53, pRb, E2F1, bax, and caspase 3 in these diseased neurons [134].

Defective DNA damage repair mechanisms in motor neurons have been suggested to underlie cell death in ALS, accelerating the disease pathology [78]. An inhibitor of DNA damage induced apoptosis and promoter of cell survival- Human Speedy A1 (Spy1) was shown to have decreased expression levels in ALS motor neurons in both in vivo and in vitro conditions promoting DNA damage response and apoptosis [176]. Elevated p53 levels have also been identified in the motor neurons of CNS tissues from ALS patients [135]. Mutations in gene encoding Cu/Zn superoxide dismutase 1 (SOD1) have been extensively reported to be associated with ALS pathology [177] and irregular CDK5 activity in association with p25 was identified in SOD1G37R mutant mice. Increased p25 levels were evident in the mutant model compared to control mice and this paralleled with aberrant CDK5 localization in the motor neurons of SOD1 mutant mice [136]. Nuclear localization of CDK4 in the spinal motor neurons of SOD1 mice compared to control mice were also reported with low levels of CDK6 but not CDK2. High cyclin D1 expression was observed in the nuclear fractions in SOD1 mutant mice unlike cyclin D2 and cyclin D3 proteins, which were mostly limited to the cytoplasm of spinal motor neurons [137]. ALS pathology and relation to cell cycle events is also observed with respect to miRNAs that act upstream to gene regulation. Altered expression profile of cell cycle regulating miRNAs were identified in brainstem motor nuclei and primary motor cortex of aged G93A-SOD1 ALS mice model [178].

### Parkinson's disease

PD is a neurodegenerative disorder charecterized by loss of control over body movements. The inadequate production of neurotransmitter dopamine and the deterioration of dopamine producing cells in the brain is the primary cause of PD. Progressive loss of dopamine-producing neurons in the substantia nigra (SN) is one of the main features of PD pathology and the disease witnesses the presence of several hallmarks of deregulated cell cycle events. Autopsy tissues from PD patients have revealed hyperphosphorylated Rb in both SN nuclei and hippocampus [138]. Aberrant cell cycle activation was evident in SN with the typical characteristics of actively replicating cells demarcated by increased DAPI staining, PCNA and E2F positive cells. Embryonic rat midbrain neurons exposed to 1-methyl-4-phenylpyridinium (MPP+), a toxic metabolite of neurotoxin MPTP (1-methyl-4-phenyl-1,2,3,6-tetrahydropyridine) depicted G1-M phase markers manifesting increased cyclin D, cyclin A, cyclin E and cyclin B levels. Remarkably, these cells showed hyperphosphorylated Rb and an increase in the transcription of E2F target genes suggesting an underlying active DNA replication in the degenerating neurons. This unusual cell cycle activation-induced apoptosis phenomenon was supported by evidence of colocalization of caspase-3 in BrdU positive cells [139]. CDK5 and CDK2 also contribute to neuronal loss in PD. Accordingly, dopaminergic neurons undergoing degeneration in the substantia nigra pars compacta of MPTP treated mice had increased CDK5 and CDK2 levels. Further, Flavopiridol, a CDK inhibitor reversed the nigral degeneration effects associated with CDK5 [140].

Alpha-synuclein accumulation in Lewy Bodies is a hallmark biochemical feature of PD brain [38]. The protein was shown to play a protective role in PD pathology by regulating cell cycle events [179]. Wildtype α-synuclein effectively restricted p53 mediated apoptosis in both neuronal and HEK293 cells and these effects were reversed upon treatment with dopaminergic neurotoxin, 6-hydroxydopamine. There was also a concomitant decrease in p21, a downstream target of p53 and reduced caspase 3 activity in these cells [179, 180]. α-synuclein mediated cyclin B accumulation has also been observed in Lewy bodies in PD and dementia [181]. An association between cyclin-G-associated kinase (GAK) gene and PD was identified in GWAS study by Dumitriu et al. (2011). Primary rat midbrain neuronal cultures overexpressing α-synuclein or exposed to A53T mutant α-synuclein, were more prone to cell toxicity effects when deprived of GAK [182]. Interestingly, genome-wide association studies in the Han Chinese PD subjects revealed association of multiple SNPs of GAK in PD pathogenesis [183]. Another report exploring the connection between GAK rs1564282 C/T polymorphism and PD pathology confirmed these findings in a meta-analysis carried out in a larger group of 8159 PD patients and 12,747 controls. Together, these reports suggest that both Asian and Caucasian populations having this SNP were potentially at a higher risk to develop PD [184]. Further, elevated p53 protein levels were observed in the caudate nucleus whereas no significant differences were observed in the dopaminergic neurons in other regions of the post-mortem brain obtained from PD subjects [141]. Oxidative stress induced by 6-hydroxydopamine in a cellular model mimicking PD pathology also exhibited enhanced p53 activation in the dopaminergic cells. Parkin is a p53 repressor, and its nitrosylation by nitric oxide synthase renders the protein non-functional thereby promoting p53 mediated apoptotic pathways. p53 upregulation in PD post-mortem brains indeed, was accompanied by 15-fold increase in parkin S-nitrosylation levels suggesting onset of a protective mechanism in disease conditions [143].

## Cell cycle machinery targeting compounds as neuroprotective agents

Anomalous cell cycle regulation is an important contributor to neuronal cell death, which implies that drugs targeting cell cycle have emerged as an important area of research to develop neuroprotective therapies. Cell cycle regulating compounds are anti-proliferative agents that result in cell cycle arrest. In the post-mitotic neuronal cells, however, cell cycle modulating agents can restrict unregulated nucleic acid duplication events in the absence of cytokinesis. Pharmacological inhibitors of cell cycle flavopiridol, roscovitine and olomoucine were observed to suppress DNA damaging agent, etoposide-induced apoptosis in rat primary cortical neurons [114]. Wide-spread cell cycle stimulation and associated neuronal death was observed in rat brains subjected to traumatic injury [185, 186]. Flavopiridol treatment interestingly, could attenuate cyclin D1 expression and consequently cell cycle activation in neurons and glia in brains of these animals. Improvement in cognitive and motor functions was also observed suggesting neuroprotective effects of the drug [114]. Roscovitine treatment, similarly, could rescue traumatic brain injury induced neurodegeneration in mice cortical tissue by attenuating both cyclin A and D1 expression and subsequent activation of cell cycle processes [187].

Kanungo et al. (2009) developed a CDK5 inhibitory peptide; CIP that specifically inhibited CDK5/p25 activity without affecting the normal CDK5/p35 pathway [188]. Calpain inhibition by CIP could protect mouse neurons from neurotoxic stress. Rat hippocampal slices when treated with roscovitine demonstrated improved excitatory postsynaptic potentials. A significant increase in glutamate release from synaptosomes and enhanced P/Q-type voltage-dependent calcium channel (VDCC) activity was also observed suggesting that CDK5 inhibition could promote neurotransmitter release and neurotransmission [189]. Neuronal progenitor cells overexpressing p35 and Aβ peptide to imitate Alzheimer’s like characteristics exhibited aberrant CDK5 activation which was rescued upon roscovitine treatment. Corroborating the cellular observations, neuroprotective effects of roscovitine were also evident in APP/PS1 transgenic mouse model of AD [190]. Similarly, spinal cord injury induced cell cycle activation and associated neuronal apoptosis and behavioural symptoms in rats could be rescued by olomoucine treatment by suppressing elevated cell cycle proteins cyclin A, cyclin B, cyclin E and PCNA. Olomoucine was also shown to be effective in suppressing astroglial proliferation, neuronal apoptosis and improving behavioural outcomes. Cell cycle inhibition has further been suggested to promote axonal regeneration in post-traumatic stages of CNS injury [191]. Yang et al. (2013) demonstrated the use of kenpaullone as a protective agent for human pluripotent stem cells derived from ALS subjects. Kenpaullone is a potent CDK1/cyclin B inhibitor that was found to specifically inhibit GSK3β [192]. Another potent inhibitor of GSK3β and CDK5/p25 named indirubin, an indole derivative constitutes an ingredient of Chinese traditional medicine for leukemia treatment. Phosphorylation of recombinant human tau and DARPP-32 by GSK3β and CDK5 respectively was diminished by Indirubin treatment [193].

Bexarotene, a Retinoid X receptor (RXR) agonist is used in cutaneous T cell lymphoma management. This drug activates ataxia-telangiectasia mutated protein ATM and downstream p53 mediated targets of cell cycle arrest and apoptosis [194]. This drug has been shown to exhibit neuroprotective properties in mice models of AD upto certain extent. Our studies identified that the drug was able to rescue the RGC degeneration and apotosis in two different glaucoma animal models [195]. The drug treatment also resulted in enhanced cell survival, neuritogenesis, reduced ER stress response and apoptotic pathway suppression in SHSY5Y neuroblastoma cells in a dose dependent manner [196]. It is to be acknowledged that use of cell cycle inhibitors may have characteristic pharmacological drug limitations of non-specific actions on structurally related non-cell cycle kinases. Further development and *in vivo* validation of compounds that can specifically modulate various cell-cycle regulatory targets will help identify novel avenues for neuroprotection [197, 198].

**Figure 2.**
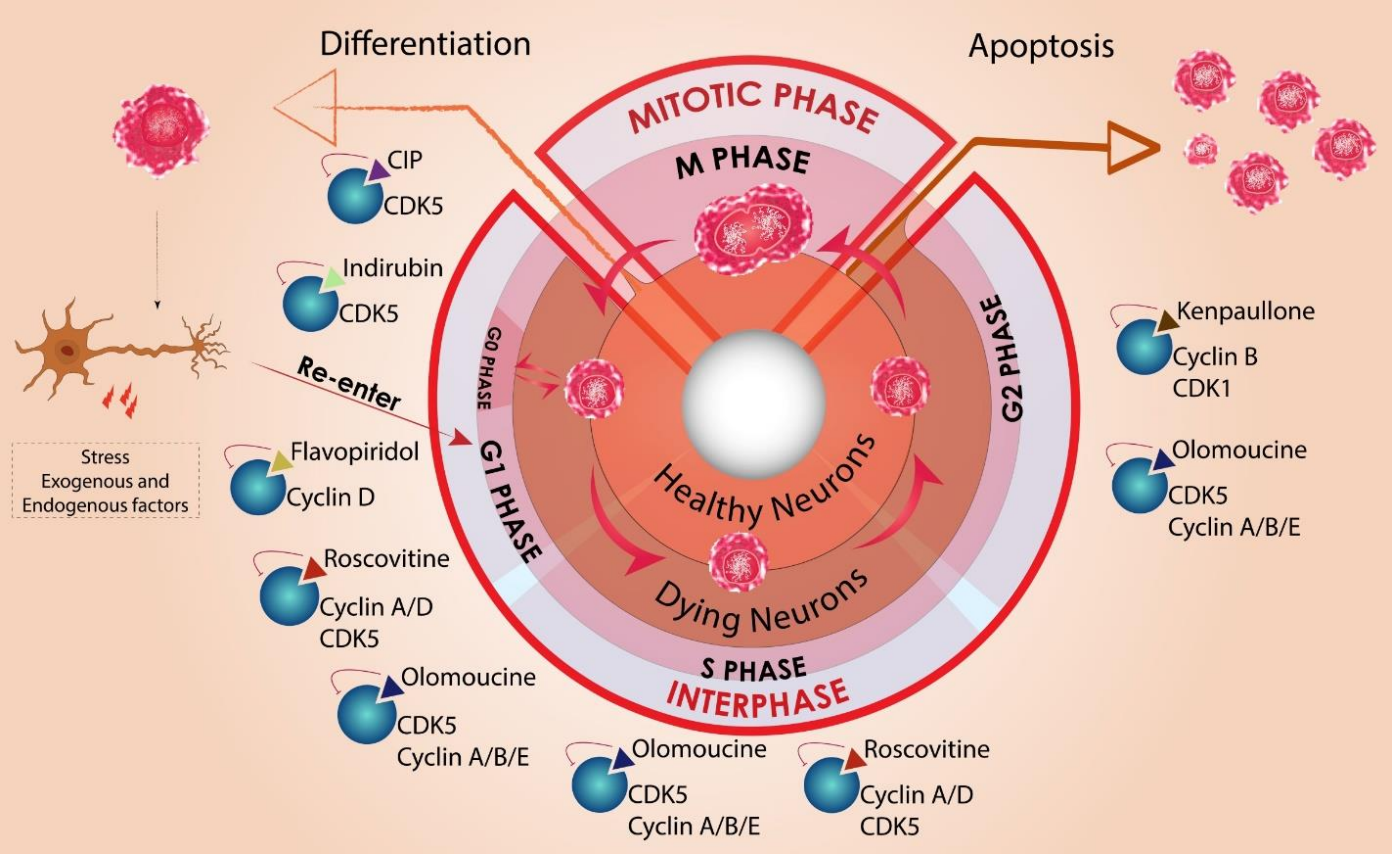
**Schematic representation of cell cycle in healthy and degenerating neurons**. In physiological conditions neural precursor cells go through G1, S, G2 and mitotic phases of cell cycle (inner circle) delivering early progenitor cell that can differentiate to mature neurons (left arrow). However, a mature dormant neuron in G0 phase may re-enter the cell cycle under pathological conditions. This cell then proceeds through all the different phases in interphase but is hindered at the end of G2 (outer circle). Instead of proceeding to mitotic (M) phase these tetraploid cells may undergo apoptosis (right arrow). Pharmacological compounds that affect various cell cycle stages and potentially could be neuroprotective at different stages of the cell cycle are also shown. Cyclin-dependent kinase (CDK5) and cyclin D in G1 phase can be inhibited by CDK5 inhibitory peptide (CIP), indirubin, and flavopiridol respectively. Roscovitine inhibits CDK5 interaction with multiple cyclins involved in G1 and S phase. Olomoucine imparts neuroprotection by regulating CDK5/cyclin interaction throughout interphase. Kenpaullone is a potent cdk1/cyclin B inhibitor effective in G2 phase.

## Conclusions and future prospects

Anomalous regulation of cell cycle and consequent failure of mitotic compliance is increasingly believed to contribute to neurodegenerative changes in ageing and disease. Neurons under physiological conditions express cell cycle regulating proteins, but understanding how their expression, post-translational modifications and sub-cellular localisations are altered, will reveal their mechanistic involvement in neuropathological conditions. Amonsgt several factors discussed above, protein aggregation and asociated neurotoxicity has been suggested to increase the pre-disposition of cells towards dysregulated cell-cycle in neurodegenerative disorders. Oxyradicals generated from oxidative stress similarly can damage DNA and induce DNA damage response mechanisms that may trigger cell cycle initiation events in quiescent cells. A cumulative effect of these events is modulation of essential cell cycle proteins such as cyclins, CDKs and CDKIs in neuronal cells leading to apoptosis and neuronal loss observed in various neurodegenerative disorders [133, 139].The progression of neurons through cell cycle and pharmacological modulators effective at each phase of cell cycle are illustrated in Figure 2.

Although there is sufficient evidence to suggest that cell cycle entry eventually triggers neuronal death there are missing links in mechanisms underlying this process [199]. The expression pattern of various cell-cycle regulatory proteins can be significantly different during the early and late stages of disease. Expression profile for cell cycle-related proteins proliferating cell nuclear antigen, cyclin D, and cyclin B in mild cognitive impairment to severe AD cases demonstrated the occurence of cell cycle events in both early and late stages of disease [108]. Transcriptomics and proteomics investigations in cells before and after entering the cell-cycle event will help unravel various biochemical pathways that are affected under such conditions [200, 201]. Advances in gene therapy using adeno-associated virus (AAV) and lentiviruses has been able to target specific proteins and pathways *in vivo* [202, 203]. For instance, cyclins, CDKs, DNA damage checkpoint kinases and PLKs are potential candidates for cancer gene therapy that can regulate uncontrolled proliferation rates of cells [204]. E2F can be another candidate for gene targeting as E2F transcription factor decoy was indeed shown to modulate cell cycle progression in rat glomerular cells *in vivo* [205]. Various growth factors and activation of their receptors as well as phosphatases and kinases regulating their downstream signalling have been extensively shown to modulate cellular survival and cell cycle events and could be promising gene therapy targets [206-208]. These genetic modulation approaches will help in specific targeting of cell cycle regulatory proteins to develop mechanism-based therapeutics and help understand the molecular basis of downstream events of cell cycle dysregulation.
